# Aster leafhopper survival and reproduction, and Aster yellows transmission under static and fluctuating temperatures, using ddPCR for phytoplasma quantification

**DOI:** 10.1038/s41598-017-18437-0

**Published:** 2018-01-10

**Authors:** Md H. Bahar, Tyler J. Wist, Diana R. Bekkaoui, Dwayne D. Hegedus, Chrystel Y. Olivier

**Affiliations:** 1 Charlottetown Research and Development Centre, Agriculture and Agri-Food Canada, 440 University Avenue, Charlottetown, PE C1A 4N6 Canada; 2 Saskatoon Research Centre, Agriculture and Agri-Food Canada, 7 Science Place, Saskatoon, Saskatchewan S7N 0X2 Canada

## Abstract

Aster yellows (AY) is an important disease of Brassica crops and is caused by *Candidatus* Phytoplasma asteris and transmitted by the insect vector, Aster leafhopper (*Macrosteles quadrilineatus*). Phytoplasma-infected Aster leafhoppers were incubated at various constant and fluctuating temperatures ranging from 0 to 35 °C with the reproductive host plant barley (*Hordium vulgare*). At 0 °C, leafhopper adults survived for 18 days, but failed to reproduce, whereas at 35 °C insects died within 18 days, but successfully reproduced before dying. Temperature fluctuation increased thermal tolerance in leafhoppers at 25 °C and increased fecundity of leafhoppers at 5 and 20 °C. Leafhopper adults successfully infected and produced AY-symptoms in canola plants after incubating for 18 days at 0–20 °C on barley, indicating that AY-phytoplasma maintains its virulence in this temperature range. The presence and number of AY-phytoplasma in insects and plants were confirmed by droplet digital PCR (ddPCR) quantification. The number of phytoplasma in leafhoppers increased over time, but did not differ among temperatures. The temperatures associated with a typical crop growing season on the Canadian Prairies will not limit the spread of AY disease by their predominant insect vector. Also, ddPCR quantification is a useful tool for early detection and accurate quantification of phytoplasma in plants and insects.

## Introduction

Aster yellows (AY) disease in Brassica and in numerous other crops in North America has received considerable attention because of its significant negative economic impact on agriculture. AY is caused by a phytoplasma of the taxon ‘*Candidatus* Phytoplasma asteris’^1^ which is a cell-wall-less bacterium transmitted primarily by phloem-feeding leafhoppers^2^. While there are more than 20 leafhopper species known to spread phytoplasma diseases, the Aster leafhopper, *Macrosteles quadrilineatus* Forbes (Hemiptera: Cicadellidae), is the major vector of AY-phytoplasma in Brassica crops^3–5^.

Biological parameters such as physiology, biology and behaviour of insects, pathogens and plants, will be affected by global climate change^6^. For example, the distribution of many insects and pathogens is shifting toward the Earth’s poles^7^. Furthermore, rising temperatures could increase the possibility of new introductions of vectored phytoplasma diseases^8^. To predict future epidemiological performance and distribution of the AY-phytoplasma along with its vector insects and host plants, detailed studies on the impacts of variable temperatures on the whole disease system are crucial. However, the impact of variable temperatures on phytoplasma epidemiology has received little attention. A few studies have evaluated thermal effects on phytoplasma, vector insects and plants separately, but these have several limitations. The effect of temperature on the transmission of ‘*Ca*. Phytoplasma asteris’ by *Macrosteles quadripunctulatus* Kirschbaum was investigated at only four constant temperatures^9^, while another study used two constant temperatures to report that the multiplication kinetics of *Flavescence doree* doubled at 25 °C compared to 20 °C^10^. Nevertheless, experiments that incorporate incremental changes in temperature are more ecologically relevant than those using constant temperatures and ensure that the studied species have time to express their physiological coping mechanisms^11^.

Studies at constant extreme temperatures versus fluctuating temperature showed differences in the thermal limits of insects^12,13^. A variable environment may result in different natural patterns than are evident under the average environmental conditions predicted by Jensen’s inequality theory^14^. The sooty copper butterfly, *Lycaena tityrus* (Lepidoptera: Lycaenidae), develops faster at fluctuating temperatures than it does at constant temperatures^15^. Also, the development time of the diamondback moth, *Plutella xylostella* (Lepidoptera: Plutellidae), followed the same pattern of shorter development time at temperatures that fluctuate around the mean (0–14 °C) than at a constant mean of 7 °C^13^. Experiments with fluctuating as opposed to constant temperatures may be a more accurate way to assess the effect of temperature on the biology of insects or pathogens^16^. Another potential limitation of other studies is the investigation of a single organism instead of the complex of organisms that are involved in the epidemiology of a disease. Parasites and their hosts often exist in a balanced equilibrium of population densities and their resulting disease epidemiology may also be at equilibrium^17,18^. Provided that synchrony between the development of a parasite and its host is affected by temperature, changing temperatures may be more beneficial to one species than another. Hence, the synchrony between phytoplasmas and their occurrence in an insect vector may be influenced by climatic factors^19^. Other studies have investigated the effect of temperature on phytoplasma and its host plant^11^, and phytoplasma and its insect vector^19^; however no study has investigated thermal effects on a whole disease system comprising phytoplasma, leafhopper and the host plant.

Various techniques, including ELISA, PCR and RT-qPCR, are used to quantify and monitor the distribution and movement of phytoplasmas in plants^20–22^. Droplet digital PCR (ddPCR) technique is becoming more widely used to quantify microorganisms by determining the number of specific genetic markers^23–27^. Relative to other PCR techniques, ddPCR provides advantages in dynamic range, including detection of very low concentration of target molecules in a sample, with a reduced per sample cost^28,29^. Unlike analog quantitative PCR (qPCR) where a standard curve is used to estimate target concentration, ddPCR is an endpoint and absolute measurement approach through which target copy number can be determined using the Poisson distribution^29^. Incidentally, ddPCR is more sensitive than qPCR for the detection of rare target molecules and more accurate at low target copy numbers^23^. The use of ddPCR as a tool to quantify phytoplasma was validated by Mehle *et al*.^30^, who quantified *Flavescence dorée* phytoplasma in grapevine by estimating the numbers of phytoplasma per µL of DNA. Perez-Lopez *et al*.^31^ also used ddPCR to quantify phytoplasmas in berries and periwinkle plants. However, no internal control assays were used in these studies. No studies exist on the quantification of phytoplasmas in a vector insect using ddPCR.

The objectives of this study were to investigate: (1) the survival and multiplication of the AY-phytoplasma; (2) the survival, reproduction and disease transmission capacity of its insect vector *M. quadrilineatus* and (3) the survival of its plant food and reproductive host, barley (*Hordeum vulgare* L) (Poaceae) through a range of constant and variable temperatures which simulated a natural climate using a unique thermal gradient cell array. The number of AY-phytoplasma present in the plants and insects were quantified using ddPCR allowing absolute quantification^23^.

## Results

To assess the effects of temperature, AY-phytoplasma infected and healthy leafhoppers on barley plants were incubated at different constant (0, 5, 10, 15, 20, 25, 30 and 35 °C) and fluctuating temperatures (0–10 °C with a mean of 5 °C, 15–25 °C with a mean of 20 °C, 20–30 °C with a mean of 25 °C, and 25–35 °C with a mean of 30 °C) in individual cells of a thermal gradient system. The survival and reproduction of leafhopper, and the numbers of phytoplasma in insect and plant are presented in this section.

### Effect of temperature on leafhopper survival

There was a significant effect (*P* < 0.001) of temperature on the survival of leafhoppers by the 7^th^ day, but AY-infection had no effect on leafhopper survival (*P* = 0.10), with no significant interaction between AY-infection and time (*P* = 0.12). Similarly, on the 14^th^day, there was no significant interaction between AY-infection and time (*P* = 0.10) and no effect of AY on the survival of leafhoppers (*P* = 0.50), but there was a significant effect (*P* < 0.001) of temperature on leafhopper survival. This trend continued until the 18^th^ day, with no significant interaction between AY-infection and time (*P* = 0.10), and no effect of AY-infection on survival (*P* = 0.50), but a significant effect (*P* < 0.001) of temperature on leafhopper survival (Table 1). On all three sampling days, the highest percentages of surviving leafhoppers were found in assays occurring at constant temperatures of 5, 10, 15, 20 °C and at fluctuating temperatures between 5 and 20 °C. After 18 days, no leafhoppers survived at a constant temperature of 35 °C or a fluctuating temperature with a mean of 30 °C (Table 1). Overall, temperature fluctuation did not have a significant effect (7^th^ day *P* = 0.88; 14^th^ day *P* = 0.92; 18^th^ day *P* = 0.96) on the survival of leafhoppers, with the exception of 25 °C on the 14^th^ and 18^th^ days, where significantly more leafhoppers survived at the fluctuating temperature regime than the constant regime (Table 1).

**Table 1:** Percentage surviving leafhopper adults infected by AY-phytoplasma(AY + LH) or un-infected (AY − LH) after 7, 14 and 18 days of incubation at various temperatures.

Infection status of leafhoppers (LH)	Surviving (% ± SE) LH adults after 7, 14 and 18 days of incubation at various temperatures											
	Const 0 °C	Const 5 °C	Const 10 °C	Const 15 °C	Const 20 °C	Const 25 °C	Const 30 °C	Const 35 °C	Fluc 5 °C	Fluc 20 °C	Fluc 25 °C	Fluc 30 °C
After 7 days												
AY + LH	42 ± 3.4b	67 ± 6.0a	71 ± 6.8a	77 ± 4.4a	63 ± 6.4a	39 ± 9.7bc	35 ± 4.2bc	3 ± 3.0d	71 ± 7.5a	56 ± 6.0ab	46 ± 2.9b	27 ± 7.5c
AY − LH	43.3 ± 6.7b	73.3 ± 7.3a	75 ± 5.8a	60 ± 18.0a	60 ± 7.6a	8.3 ± 8.3d	28.3 ± 3.3c	6.7 ± 6.7d	85 ± 2.9a	51.7 ± 8.3ab	23.3 ± 11.7c	23.3 ± 6.7c
After 14 days												
AY + LH	20 ± 5.7b	52 ± 4.6a	35 ± 3.16a	33 ± 6.4a	27 ± 11.0ab	5 ± 5.0c	9 ± 2.4c	0 ± 0d	53 ± 9.0a	17 ± 8b	13 ± 7.0b	7 ± 3.0c
AY − LH	36.7 ± 4.4c	53.3 ± 1.7b	41.7 ± 10.9bc	33.3 ± 9.2b	10 ± 10de	0 ± 0f	3.3 ± 1.6e	3.3 ± 3.3e	70 ± 7.6a	15 ± 2.9d	6.7 ± 3.0e	5 ± 5.0d
After 18 days												
AY + LH	12 ± 5.1b	32 ± 4a	18 ± 4ab	7 ± 3.0b	10 ± 6.0b	1 ± 1.0c	2 ± 2c	0d	30 ± 4.7ab	9 ± 6.5b	7 ± 5.8b	0d
AY − LH	20 ± 2.8b	35 ± 2.8a	18.3 ± 9b	15 ± 10b	5 ± 5.0b	0c	0c	0c	48.3 ± 6.0a	0c	0c	0c

Means with same letters within a row are not significantly different (*P* ≤ 0.05, Tukey’s HSD).

### Effect of temperature on leafhopper reproduction

**Figure 1:**
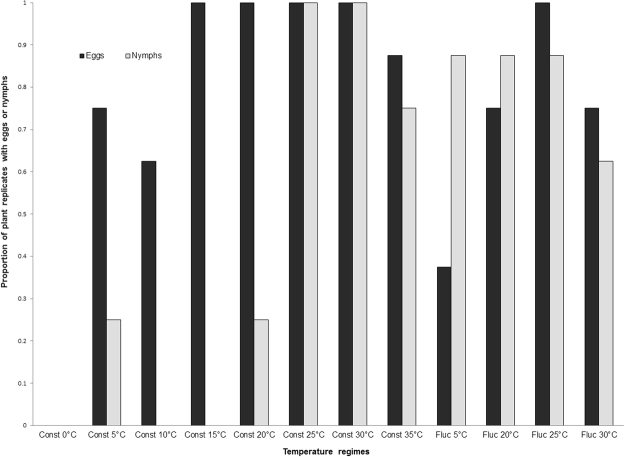
Proportion of barley plants (N = 8 per temperature treatment) with eggs or nymphs of Aster leafhopper after 18 days of incubation at various constant (Const) and fluctuating (Fluc) temperatures (°C).

Some leafhopper adults survived at a constant temperature of 0 °C, but did not reproduce as no eggs or nymphs were found on the barley plants. At 35 °C, no adults survived after 18 days, but leafhopper eggs and nymphs were observed on 70% of the barley plants. At constant temperatures of 5, 10, 15 and 20 °C, eggs were found on 60–100% of the barley plants, but very few nymphs were observed (Fig. 1). Temperature fluctuation had measurable effects on egg and nymph production at 5, 20 and 30 °C. More plants had eggs at constant temperatures, whereas more plants had nymphs at fluctuating temperatures of 5 and 20 °C averages. Fewer plants had eggs and nymphs at a fluctuating temperature with a 30 °C mean than a constant temperature of 30 °C (Fig. 1).

### Detection of AY-phytoplasma in barley plants

Barley plants infested with either AY-infected or -uninfected leafhoppers survived at all temperatures except 35 °C; however, phytoplasma were not detected in any of the barley plants.

### Detection and quantification of AY-phytoplasma in Aster leafhopper

**Figure 2:**
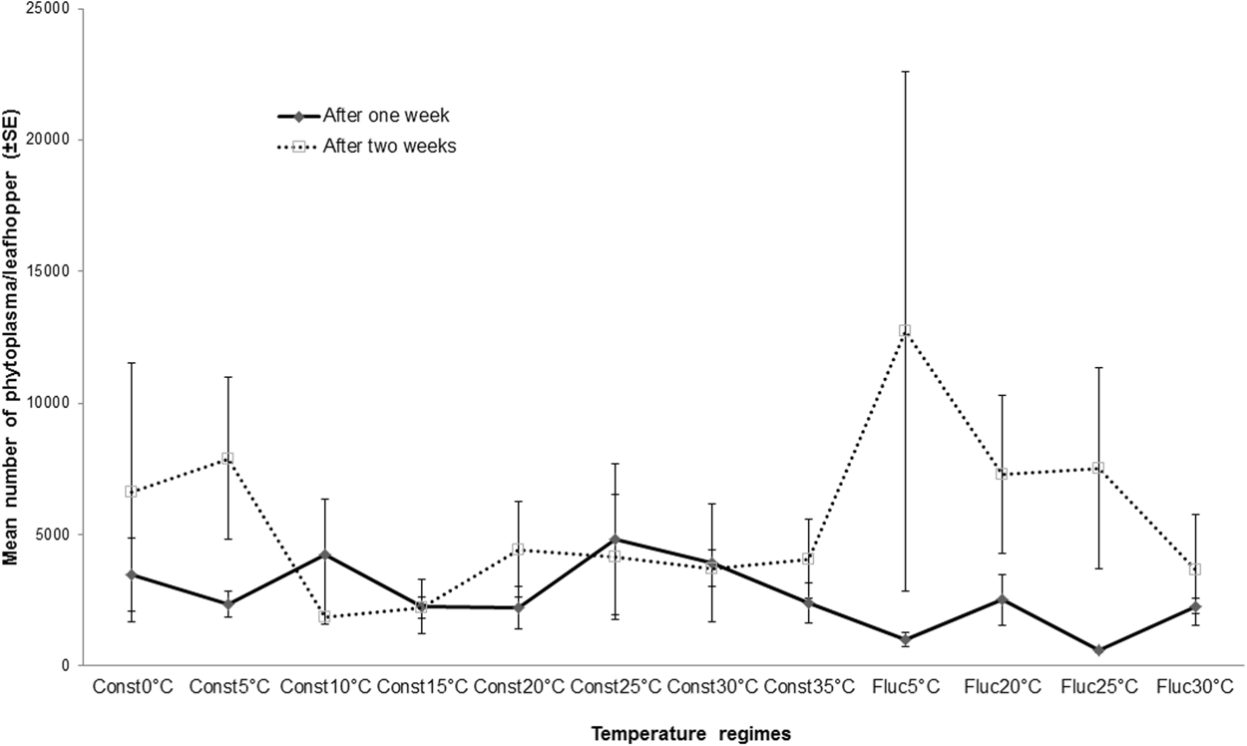
Mean numbers of phytoplasma (±SE) present in each adult leafhopper after incubation at various constant (Const) and fluctuating (Fluc) temperatures for one week and two weeks, quantified by ddPCR.

There was no interaction (*P* = 0.25) between time, temperature fluctuation and temperature on the mean number of phytoplasma present in a single leafhopper. There was a significant increase in the number of AY-phytoplasma in leafhoppers after two weeks of incubation compared to one week of incubation (*P* = 0.005). However, there was no difference (*P* = 0.96) in phytoplasma number among the temperatures (Fig. 2).

### AY-phytoplasma in canola plants

**Figure 3:**
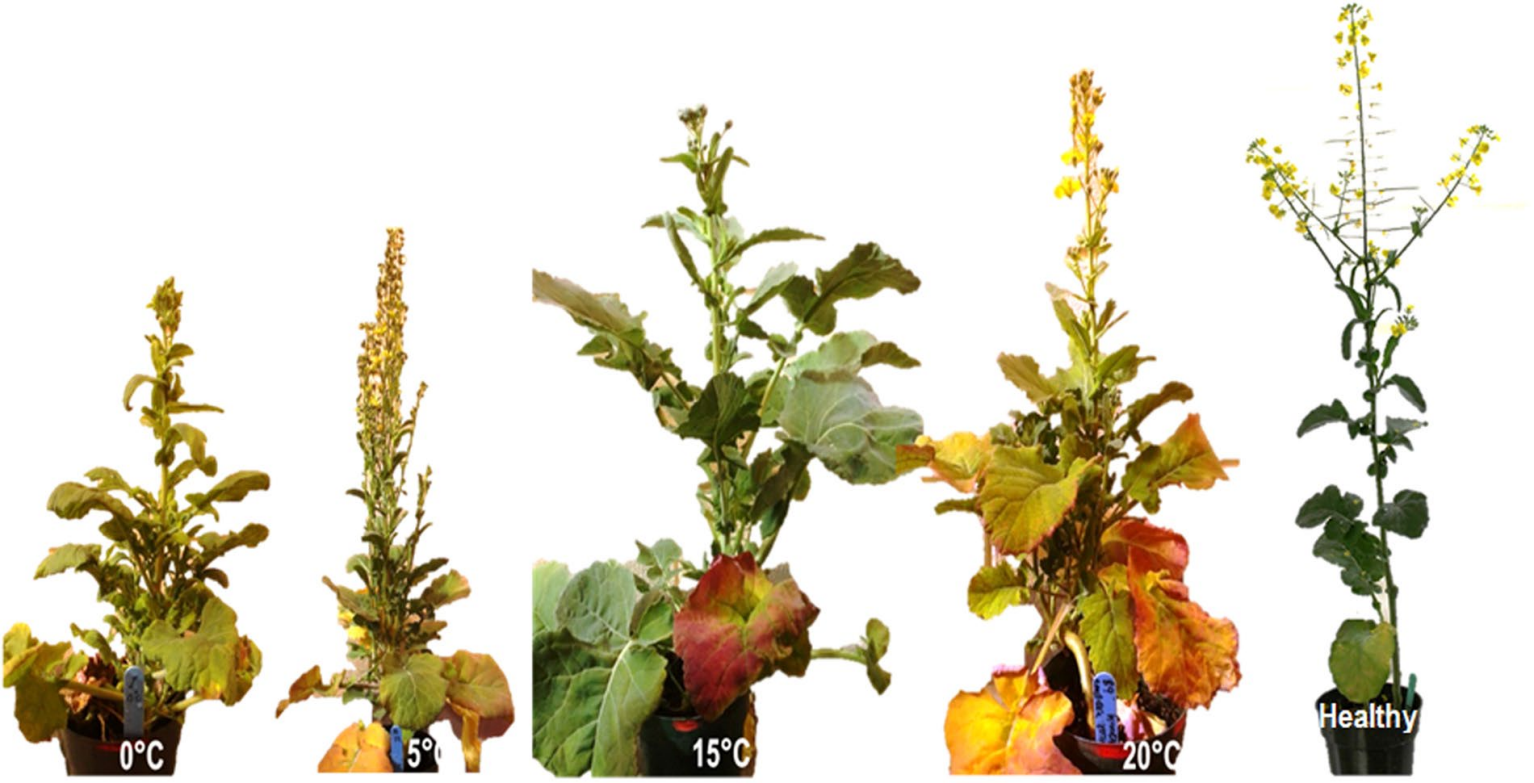
Typical Aster yellows symptoms observed in canola plants (*Brassica napus* cv. AC Excel) comparing with a healthy matured plant. The plants were infested by AY-phytoplasma-carrying Aster leafhoppers that were incubated for 18 days at various temperatures.

**Figure 4:**
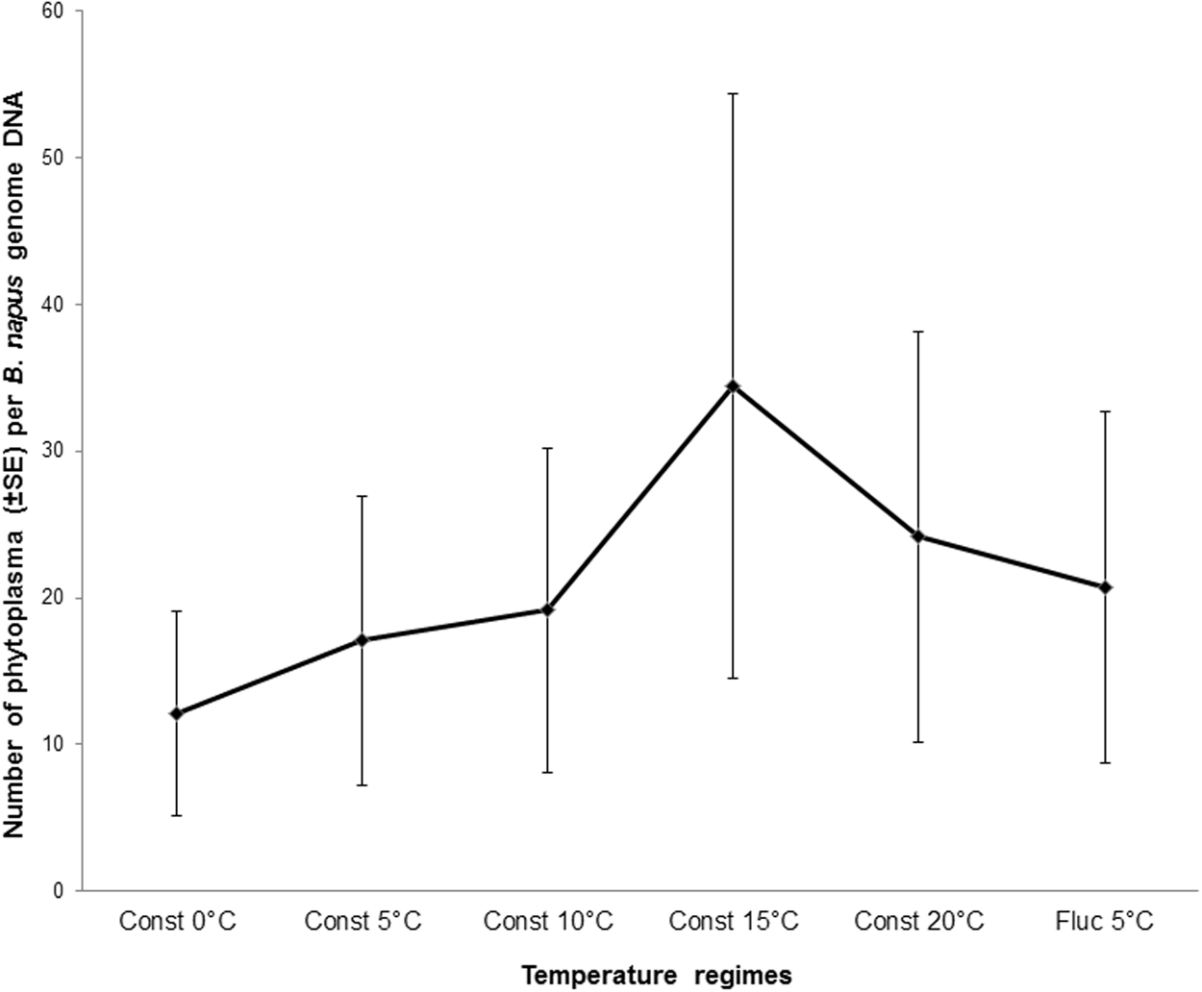
Mean numbers of phytoplasmas (±SE) present per genome DNA of canola plants (*Brassica napus* cv. AC Excel). The plants were infested by AY-leafhoppers that were incubated at five constant (Const) and one fluctuating (Fluc) temperatures.

After 18 days of incubation, AY-infected surviving leafhopper adults recovered from bioassay chambers at constant temperatures of 0 to 20 °C, as well as at a fluctuating temperature with a mean of 5 °C, were transferred to healthy *B. napus* plants. After six weeks, all plants showed symptoms typical of AY disease (Fig. 3). The presence of AY-phytoplasma in those plants was confirmed by ddPCR. There was no significant differences (*P* = 0.47) in the number of phytoplasma in plants infested with the leafhoppers incubated at various temperatures (Fig. 4).

## Discussion

In this study, we used a range of constant and fluctuating temperatures that the causal agent of Aster yellows disease, AY-phytoplasma, the vector, Aster leafhopper, *M. quadrilineatus*, and its host plants would encounter during a typical growing season on the Canadian prairies^32^. This study represents the first use of ddPCR to quantify the number of AY-phytoplasma in plants (*B. napus* and *H. vulgare*) as well as in the insect vector *M. quadrilineatus*. In addition to phytoplasma detection, the ddPCR assay allows for absolute quantification of the number of AY-phytoplasma in an individual leafhopper specimen and in plants. This quantitative information is crucial for determining the titre of AY-phytoplasma per leafhopper, which signifies the capacity for transmitting phytoplasma to plants. With the *B. napus* ddPCR primers/probe proven to function here, the absolute quantification of AY-phytoplasma from samples of canola plants is also possible. This information will help to determine to what degree AY-symptom development in canola is related to the AY-phytoplasma present in affected plant tissues. This will allow for predictions of AY-symptoms and impact on yield. The results presented here allowed the evaluation of the replication of AY-phytoplasma in both the Aster leafhopper and two host plants, over time and over a wide range of constant and fluctuating temperatures.

Temperature had a significant effect on the survival of leafhopper adults and on their reproductive capacity. Very few leafhoppers survived at temperatures ≥30 °C, while 20% of the leafhoppers survived at 0 °C over 18 days. This indicates that the upper thermal limit for Aster leafhopper survival is approximately 30 °C. A preliminary experiment also indicated that Aster leafhoppers can survive freezing temperatures; however, in this study the lower thermal limit for Aster leafhopper was not evaluated. Viable eggs, subsequently hatched into nymphs, were observed on barley plants grown under a constant temperature of 35 °C, but not at 0 °C. A greater number of eggs were observed on plants grown under constant temperatures of 5, 10, 15, 20 or 25 °C, as compared to ≥30 °C. Thus, the most suitable temperature range for Aster leafhopper survival and reproduction is 5 to 20 °C. Interestingly, the Aster leafhopper is capable of laying viable eggs even at 5 °C, which contrasts with the potato leafhopper, *Empoasca fabae* (Harris), another leafhopper that migrates into Western Canada, which does not oviposit below 9 °C^33^.

A trend toward better fitness was observed at fluctuating temperatures compared to constant temperatures. For example, the incidence of egg hatches increased at fluctuating temperatures with a mean of 5 and 20 °C, as compared to constant temperatures of 5 and 20 °C. At a constant temperature of 30 °C, all plants had eggs and nymphs, but at temperatures fluctuating around 30 °C the proportion of plants with eggs and nymphs decreased, suggesting that the upper thermal tolerance for Aster leafhopper reproduction is between 31 and 35 °C. The current study also showed that leafhopper eggs start hatching within 18 days at a temperature of 20 °C and above, which is consistent with the findings of Falzoi *et al*.^34^, who reported that the optimum temperature for egg hatching of the American grapevine leafhopper, *Scaphoideus titanus* (Hemiptera: Cicadellidae) was 22 °C.

Temperature significantly affected leafhopper survival, but not AY-phytoplasma replication in the leafhopper. On all three sampling days, the highest percentages of surviving leafhoppers were found in assays occurring at constant and fluctuating temperatures between 5 and 20 °C. There was a trend of increasing AY-phytoplasma numbers in leafhopper at constant 5 °C and fluctuating temperatures around 5 and 20 °C. In this study, leafhopper survival was not affected by AY-phytoplasma infection. In contrast, Beanland^35^ reported that female leafhoppers lived longer (28 days) on plants infected with AY-phytoplasma than on uninfected plants (19 days). An interaction between temperature and AY-phytoplasma infection of leafhoppers could influence survival. In other studies, survival between infected and uninfected leafhoppers did not differ at constant temperatures of 15 and 20 °C, but there was a significant increase in survival of infected leafhoppers compared to uninfected leafhoppers at 25 and 30 °C^36^. However, the opposite phenomenon occurred when *M. quadripunctulatus* was infected with Chrysanthemum yellows phytoplasma^37^. Tully *et al*.^38^ reported that most known mycoplasmas failed to survive at temperatures above 34 °C, but the current study found phytoplasma in the bodies of dead leafhoppers incubated at 35 °C. Unfortunately, the death of the leafhoppers at this temperature prevented transferring the leafhoppers to *B. napus* plants to determine if the phytoplasma was still active. Considering all life cycle parameters measured in this study the temperature range of 5–20 °C was the most suitable for Aster leafhoppers, and whenever leafhoppers survived they successfully transmitted AY-phytoplasma to healthy plants. Within this range, more eggs were laid by infected leafhoppers and more eggs hatched at the warmer temperatures. In *S. titanus*, an increase in winter temperature induces asynchrony between egg-hatching and budburst of grapevines (*Vitis vinifera*) showing that warmer temperatures, up to a critical point, accelerate egg hatch^19^.

Temperature-dependent transmission efficiency is a common feature of many vector-borne diseases^39^. In the current study, leafhoppers were able to transmit AY-phytoplasma to healthy *B. napus* plants and elicit AY-disease symptoms at all temperatures that permitted leafhopper survival. Thus, the insect host and phytoplasma shared the same level of fitness and temperature tolerance under the environmental conditions tested. Similarly, Galetto *et al*.^40^ established that environmental conditions have an effect on phytoplasma multiplication and that this effect is dependent on both the plant and insect host. In the current study, the potential disease-transferring capacity of leafhoppers could not be determined at the three temperature extremes (0, 30, 35 °C) as too few leafhoppers survived to permit transfer to *B. napus*. Further studies are recommended that begin with a larger number of leafhoppers or a reduction of the length of incubation to ensure that sufficient leafhoppers survive to be transferred to plants. In a preliminary experiment, adult *M. quadrilineatus* leafhoppers could survive for one week at temperatures as low as – 4 °C; therefore, further studies at lower extreme temperatures are also warranted to elucidate the lower threshold for AY-phytoplasma transmission by this leafhopper.

A combination of suitable temperature and wind trajectory brings leafhoppers and AY to the Canadian Prairies^41^. The crop must also be at a susceptible growth stage for infected, migrant leafhoppers to cause an economic loss through AY-phytoplasma infection^42^. Temperatures may not be favourable for leafhopper establishment in years with a long winter and a delayed spring on the prairies. If leafhoppers arrive on early southern winds during unfavourable conditions, they would be unable to survive or reproduce and, hence, would not transmit AY-phytoplasma. This situation is a reasonable explanation for the occurrence of only occasional outbreaks of AY in susceptible crops on the Canadian Prairies^43^. The information in this study enhances our understanding of Aster yellows disease epidemiology. A long winter with a delayed spring may reduce the potential for AY outbreaks for that crop growing season. Similarly, the sharp decline in leafhopper survival at high temperatures may result in a reduction in the spread of AY-phytoplasma later in the growing season under extreme heat conditions. At higher temperatures, even though leafhoppers reproduce before dying, their progeny may not survive the latent period of AY-phytoplasma amplification needed for leafhoppers to be effective vectors. The common temperature range of a typical growing season on the Canadian prairies, though, will not limit AY-phytoplasma transmission via the vector insect, Aster leafhopper.

## Materials and Methods

### Phytoplasma, vector insect and host plants

The AY-phytoplasma (‘*Ca*. P. asteris’ strain 16SrI-B) was obtained from an established culture at Agriculture and Agri-Food Canada, Saskatoon Research Centre, maintained in a colony with its vector, the Aster leafhopper, *M. quadrilineatus*, on barley and periwinkle plants (*Vinca minor* L. (Apocynaeae)) were kept in BugDorm 1 cages (Megaview Scientific). The AY-infected leafhopper colony was maintained in BugDorm cages on barley plants (reproductive host) with periwinkle acting as the reservoir host plant for the AY-phytoplasma in growth chambers (Conviron) at 24 ± 1 °C, with a 16 h L: 8 h D photo regime and approximately 55% RH. The AY-uninfected colony was maintained under the same environmental conditions but only on barley plants. On a bi-monthly basis, PCR tests were conducted to ensure that 100% of the AY-sink periwinkle plants and 95–100% of the adult leafhoppers were infected with AY-phytoplasma and that the uninfected leafhopper colony remained AY-phytoplasma free.

Barley, periwinkle and canola plants (*Brassica napus* L. cv. AC Excel) were grown in a growth chamber (temp: 20 ± 1 °C, 16 h L: 8 h D photo regime) in 15-cm-diameter plastic pots containing soil-less mix (modified after Stringham^44^). Slow release granular fertilizer (26: 13: 0) was added (2 g/L) and the soil was watered daily until seeds germinated and as needed afterwards. For *B. napus* plants, 3–5 seeds were initially planted and the healthiest seedling was allowed to grow in each pot.

### Experimental design

**Figure 5:**
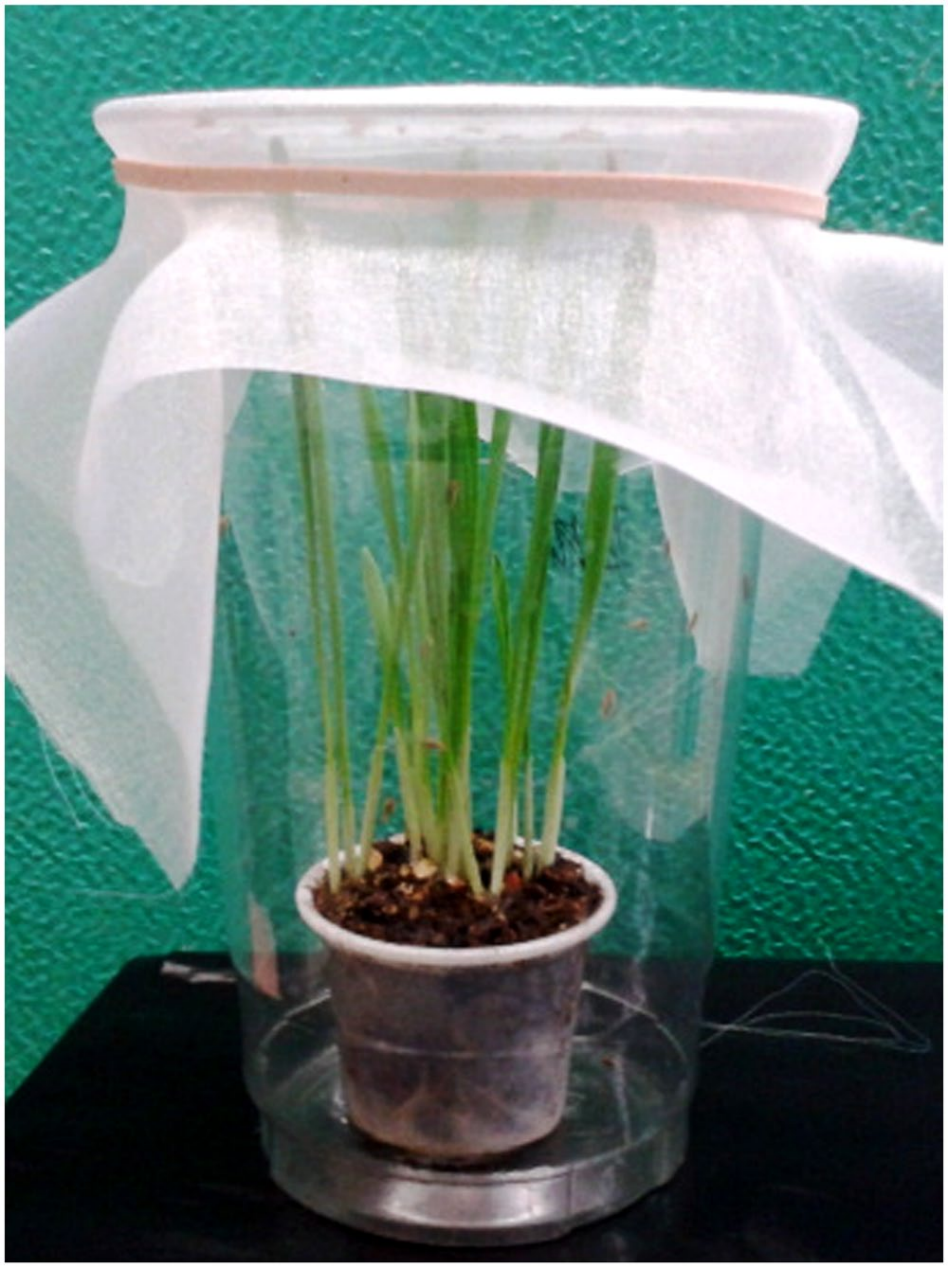
An experimental unit that was used as a whole disease system comprising AY-phytoplasma, the vector insect (Aster leafhopper) and the host plant (barley).

Each replicate consisted of 10 barley plants in a 35 ml plastic cup with 30 ml of soilless growing medium^44^. Five days after germination, each cup was placed into a 370 ml plastic transparent plastic cup and 20, 5 day-old AY-infected-adult Aster leafhoppers, collected from the colony cage using a battery operated aspirator were added in each plastic cup (Fig. 5). The plants and insects were incubated at eight different constant temperatures (0, 5, 10, 15, 20, 25, 30 and 35 °C) and four fluctuating temperatures (0–10 °C with a mean of 5 °C, 15–25 °C with a mean of 20 °C, 20–30 °C with a mean of 25 °C, and 25–35 °C with a mean of 30 °C) on a thermal gradient plate^45^. The higher fluctuating temperature phase corresponded to the 12-h light period and the cooler fluctuating temperature to the 12-h dark period. Fluctuating temperature cycles were adjusted to increase or decrease gradually by 0.5–2.0 °C/h. The thermal gradient plate had 98 individual bioassay cells (10.5 cm diam. × 12.0 cm deep) with the temperature in each cell controlled independently by a computer. Individual cell temperatures were monitored with a thermocouple and the temperature was consistently within ± 0.2 °C of the target temperature. Each cell had a glass cover to allow entry of light. Light with an intensity of approximately 110 µmole m^−2^ s^−1^ was provided by fluorescent tubes suspended 1 m above the cells. There were five replicates with AY-infected leafhopper for each temperature regime. Three control replicates were done concurrently following the same protocol with non-AY-infected leafhoppers, i.e., leafhoppers grown in a separate growth chamber on healthy barley plants. The number of surviving leafhoppers was recorded after 7, 14 and 18 days of incubation. On the 7^th^ and 14^th^ day, three leafhoppers from each replicate were collected and frozen in liquid nitrogen for DNA analysis to quantify the number of AY-phytoplasmas at each time point. Barley plants were thoroughly examined for any leafhopper eggs or nymphs and 100 mg of barley plant tissue was sampled to quantify the number of AY-phytoplasma. Each plant was counted binomially as a 1 or 0 for the presence or absence of eggs or nymphs and the proportion of plants with eggs or nymphs was calculated based on the total number of plants observed.

On the 18^th^ day, surviving leafhoppers from each temperature were transferred to three one-week old *B. napus* plants (cotyledon stage) contained in a mesh cage (BugDorm, Megaview Scientific) and allowed to feed for 48 h before being removed. The plants were maintained in a growth chamber (Conviron, model PGV 35) at 20 °C, 50–60% relative humidity, 16 h L: 8 h D photo regime and 700–800 µmole m^−2^ s^−1^ light intensity for six weeks to allow AY-symptom expression^42^. The number of *B. napus* plants expressing AY-symptoms was recorded and 100 mg of tissue from the leaf petiole of each plant was collected to quantify the number of AY-phytoplasma.

### Quantification of phytoplasma DNA using ddPCR

DNA was extracted from 100 mg of freeze-dried *B. napus* or barley plant tissue using the Plant Mini Kit (QIAGEN). DNA from single leafhoppers collected on the 7^th^ and 14^th^ days post-infestation was extracted using the Blood and Tissue Kit (QIAGEN). Approximately, 200 ng of DNA was digested with *Eco*RI (Invitrogen) in a total volume of 20 µl. ddPCR was used to quantify AY DNA in the plant or in the insect host. The phytoplasma *16S* rRNA gene was amplified from phytoplasma DNA using 2X KAPA HiFi DNA polymerase and the primers R16F2 (5′-GAAACGACTGCTAAGACTGG-3′) and R16R2 (5′-TGACGGGCGGTGTGTACAAACCCC-3′) based on the GenBank sequence (Accession DQ411470.1). The PCR product was cloned into pCR^TM^II-Blunt-TOPO (Invitrogen TOPO® DNA Cloning, Life Technologies Inc.) and several clones were sequenced. The *B. napus actin-2* gene (GenBank Accession FJ529167.1) was used as a reference to enumerate the number of AY-phytoplasma vs. plant genome equivalents. The ddPCR primers and probes for the phytoplasma *16S rRNA* and *B. napus actin-2* genes were designed using PrimerQuest software (Integrated DNA Technologies, Inc.) in accordance with the MIQE guidelines^46^ (Table 2). Phytoplasma in leafhopper was quantified using the primer and probe for *16S rRNA* and calculated as the phytoplasma copy number per leafhopper.

**Table 2:** Primers and probes designed and used in droplet digital PCR.

Gene	Direction	Sequence	Length (bp)	Tm (°C)	GC%
16S	Probe (Fam)	TATTGGGCGTAAAGGGTGCGTAGG	100	68	54.0
	Forward	GCAGCCGCGGTAATACATA		62	52.6
	Reverse	GAGCATTGCACTTAGACCATAAAC		62	41.7
Bn Actin-2	Probe (Hex)	TGCTGGATTCTGGTGATGGTGTGT	94	72	50.0
	Forward	CAGTGGTCGTACTACTGGTATTG		68	47.8
	Reverse	GATGGCGTGTGAAAGAGAGA		60	50.0

To quantify the number of phytoplasma in plants, a duplex ddPCR reaction mixture composed of 12.5 µl 2X ddPCR Super Mix for Probes, *16S* and *actin-2* primers (final concentration of each primer was 900 nM) and probe (final concentration of each primer was 250 nM), 1 µl of digested DNA (10 ng) made to a final volume of 25 µl with ddH_2_O was used. A 20 µl aliquot was used to generate droplets in an 8-well cartridge using a QX100 droplet generator (Bio-Rad, Pleasanton, CA). Droplets were transferred to a 96-well ddPCR plate, sealed with heat seal (Bio-Rad) and amplified in a conventional PCR thermal cycler. The thermal cycling conditions were as follows: 10 min denaturation at 95 °C, 50 cycles of a two-step thermal profile of 30 sec denaturation at 94 °C and 1 min annealing/extension at 58 °C. After amplification, the products were heated to 98 °C for 10 min to harden the droplets and then cooled to 12 °C. Droplets were quantified in a QX100 droplet reader (Bio-Rad, Pleasanton, CA). Data acquisition and analysis were performed using QuantaSoft software (Bio-Rad, Pleasanton, CA). Positive droplets containing amplification products were discriminated from negative droplets by setting the fluorescence amplitude threshold to the lowest point of the positive droplet cluster.

To quantify the copy number of phytoplasma in insects, a single-plex ddPCR was performed with a reaction mixture composed of 12.5 µl 2X ddPCR Super Mix for Probes, *16S* primers (final concentration of the primer was 900 nM) and probe (final concentration of each primer was 250 nM), 1 µl of digested DNA (10 ng) made to a final volume of 25 µl with ddH_2_O. The thermal cycling conditions were the same as the plant samples.

### Statistical analysis

To analyze the effects of temperature and AY-infection on the survival of leafhopper adults, a two-way ANOVA was performed in R^47^. Means were separated with a Tukey’s HSD test (α = 0.05). The number of phytoplasma in leafhoppers and plants was analyzed with PROC Logistic analysis using the Generalized Linear Model in SAS (SAS Version 9.1).
